# Liaisons dangereuses: cross-border gene flow and dispersal of insecticide resistance-associated genes in the mosquito *Aedes aegypti* from Brazil and French Guiana

**DOI:** 10.1590/0074-02760190120

**Published:** 2019-09-23

**Authors:** Patrícia Salgueiro, Johana Restrepo-Zabaleta, Monique Costa, Allan Kardec Ribeiro Galardo, João Pinto, Pascal Gaborit, Amandine Guidez, Ademir Jesus Martins, Isabelle Dusfour

**Affiliations:** 1 Universidade Nova de Lisboa, Global Health and Tropical Medicine Centre, Instituto de Higiene e Medicina Tropical, Lisboa, Portugal; 2 Institut Pasteur de la Guyane, Vectopole Amazonien Emile Abonnenc, Vector Control and Adaptation Unit, Cayenne, France; 3 Fundação Oswaldo Cruz-Fiocruz, Instituto Oswaldo Cruz, Laboratório de Fisiologia e Controle de Artrópodes Vetores, Rio de Janeiro, Brasil; 4 Instituto de Pesquisas Científicas e Tecnológicas do Estado do Amapá, Amapá, Brasil; 5 Institut National de la Recherche Scientifique, Centre Armand Frappier Santé Biotechnologie, Laval, QC, Canada

**Keywords:** Aedes aegypti, Brazil, French Guiana, insecticide resistance, population genetics, vector control

## Abstract

**BACKGROUND:**

In recent years, South America has suffered the burden of continuous high impact outbreaks of dengue, chikungunya and Zika. *Aedes aegypti* is the main mosquito vector of these arboviruses and its control is the only solution to reduce transmission.

**OBJECTIVES:**

In order to improve vector control it is essential to study mosquito population genetics in order to better estimate the population structures and the geneflow among them.

**METHODS:**

We have analysed microsatellites and knockdown resistance (*kdr*) mutations from a trans-border region in Amazonia between the state of Amapá (Brazil) and French Guiana (overseas territory of France), to provide further knowledge on these issues. These two countries have followed distinct vector control policies since last century. For population genetic analyses we evaluated variability in 13 well-established microsatellites loci in *Ae. aegypti* from French Guiana (Saint Georges and Cayenne) and Brazil (Oiapoque and Macapá). The occurrence and frequency of *kdr* mutations in these same populations were accessed by TaqMan genotype assays for the sites 1016 (Val/Ile) and 1534 (Phe/Cys).

**FINDINGS:**

We have detected high levels of gene flow between the closest cross-border samples of Saint-Georges and Oiapoque. These results suggest one common origin of re-colonisation for the populations of French Guiana and Oiapoque in Brazil, and a different source for Macapá, more similar to the other northern Brazilian populations. Genotyping of the *kdr* mutations revealed distinct patterns for Cayenne and Macapá associated with their different insecticide use history, and an admixture zone between these two patterns in Saint Georges and Oiapoque, in accordance with population genetic results.

**MAIN CONCLUSIONS:**

The present study highlights the need for regional-local vector surveillance and transnational collaboration between neighboring countries to assess the impact of implemented vector control strategies, promote timely actions and develop preparedness plans.

*Aedes aegypti* is involved in recurrent dengue epidemics and in recent chikungunya and Zika outbreaks that have occurred in South America.^1^ It is also the mosquito responsible for the historical epidemics of yellow fever reported in the Americas since the 17th century. Therefore, vector control measures have been applied for decades in both French Guiana and Brazil.^2^ ^,^ ^3^ In the 50s, dichlorodiphenyltrichloroethane (DDT) application had eradicated *Ae. aegypti* from French Guiana and Brazil. However, the mosquito returned a decade later resistant to this molecule. Organophosphates (OP) were then applied and replaced in the 90s by pyrethroids (PY) as adulticides and *Bacillus thuringiensis israelensis* (*Bti*) as larvicides.^2^ ^,^ ^3^

Presently, vector control is implemented in both territories by breeding sites removal or treatment all year round against larvae, spatial spraying of adulticides during outbreaks and by community engagement programs.^3^ Since 2011, deltamethrin (PY) is the only authorised and available insecticide in French Guiana for spatial sprays against adult mosquitoes.^3^ In Brazil, the choice of adulticides includes four PYs and malathion (OP).^4^ High resistance to PYs is now recorded in the two countries.^4^ ^,^ ^5^ ^,^ ^6^ PYs target the voltage-gated sodium channel of neuronal membranes of insects. Besides metabolic resistance, mutations in the gene encoding this channel have been associated with DDT/PY cross-resistance, known as knockdown resistance (*kdr*). Two main mutations are monitored and widespread in *Ae. aegypti* populations across Latin America and the Caribbean. Some publications relate that V1016I is associated with deltamethrin resistance,^7^ while F1534C is associated with permethrin and DDT ones^8^ and enhances the V1016I effect.^7^ In French Guiana, the presence of the resistance-associated *kdr* mutations V1016I and F1534C has been reported in Cayenne since 2011.^5^ In Brazil, *kdr* haplotypes differ according to geographical region, suggesting structured populations. The R1 haplotype (mutant only at position 1534) was found in the whole country, whereas R2 (mutant at both 1016 and 1534 positions) is more common in the Central and Southern region.^9^

The success of vector control campaigns is influenced by aspects like the effective size of the mosquito population, their temporal genetic stability and the connectivity between adjacent populations.^10^ Patterns of mosquito population structure are shaped by human activities coupled with environmental factors like seasonality or landscape.^11^ ^,^ ^12^ Variations in space and time of population genetic structure of *Ae. aegypti* have been studied in Brazil.^13^ ^,^ ^14^ ^,^ ^15^ In French Guiana, isozymes and RAD-Seq indicated a strong population structure in local *Ae. aegypti*.^16^ ^,^ ^17^ In Brazil, North and South populations are genetically partitioned as also found for *kdr* alleles.^13^ ^,^ ^14^ Population genetics is thus essential to unravel resistance dynamics, to understand the origin of selective pressures, the gene flow among populations and the causes of resistance maintenance in a population, helping to successfully implement insecticide resistance management plans.

The lack of knowledge in trans-border regions such as that between Brazil and French Guiana can be hazardous for control efforts implemented in both sides of the frontier. Here we have analysed microsatellites and *kdr* mutations from four *Ae. aegypti* populations from Amapá state (Brazil) and French Guiana (France), in order to: (1) decipher if insecticide resistance is due to a local selective pressure; (2) if distance and the Oyapock river act as a physical barrier for the spread of *kdr* genes and (3) if seasonal factors could influence the pattern of *kdr* frequencies.

## MATERIALS AND METHODS

**Fig. 1:**
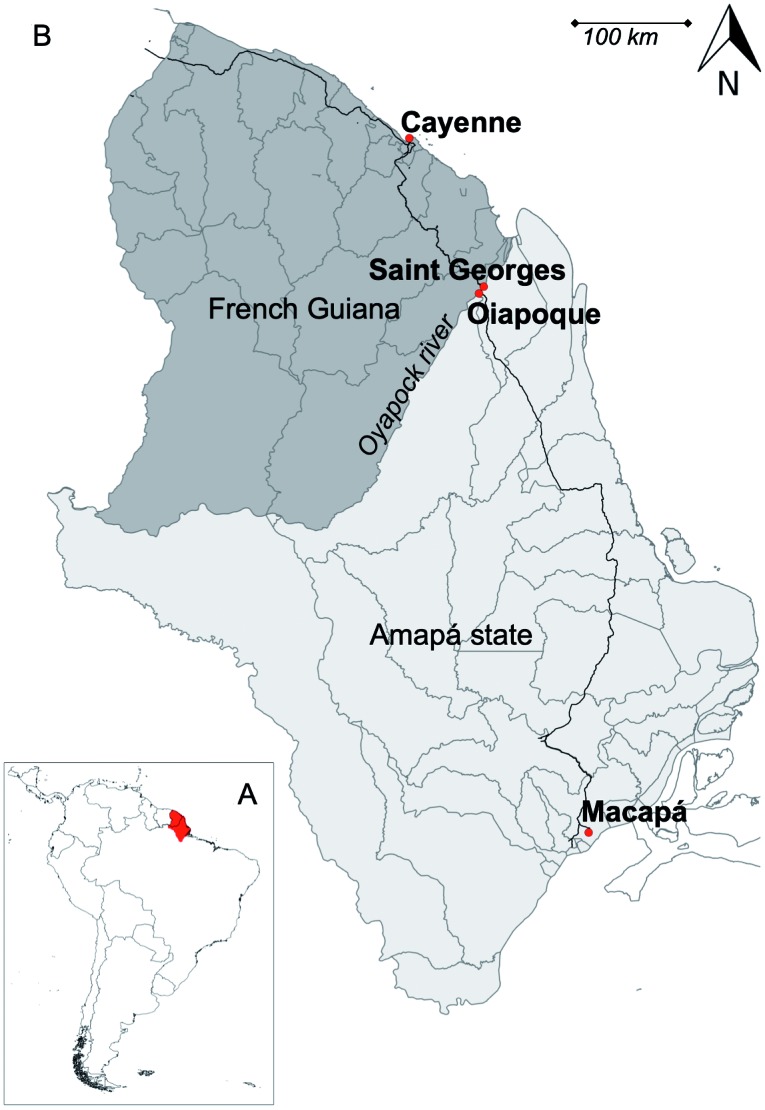
map showing the location of French Guiana and the Amapá state, Brazil in South America (A) and the four localities sampled between 2013 and 2014 for the present study i.e. Saint Georges de l’Oyapock (4,037 inhabitants) and Cayenne (59,753 inhabitants) in French Guiana ; Macapá (474,706 inhabitants) and Oiapoque (25,514 inhabitants), state of Amapá in Brazil (B).

*Study sites* - The cross-border region of Amapá state, Brazil and French Guiana, France, is situated in the North of South America (Fig. 1A). We have sampled two urban areas of French Guiana: Saint Georges de l’Oyapock and Cayenne, and other two of the state of Amapá in Brazil: Macapá and Oiapoque, (Fig. 1B). The distance between Macapá, the Amapá state capital, and Oiapoque is 589 km. Oiapoque and Saint Georges are separated by the Oyapock river, corresponding to the border between the two countries. The connection between the two is still mainly made by boat, although a 378 m long bridge has been opened in March 2017 only to passenger vehicles. Saint Georges is connected to Cayenne, the French Guiana capital, by a road of 189 km.

*Mosquito collection* - Sampling was performed during the rainy (May) and dry (December) seasons from December 2013 to December 2014 in French Guiana in Cayenne-CAY and Saint-Georges de l’Oyapock-SGO. In Brazil, collections took place in the rainy (May) and dry (December) seasons of 2014 in two locations of the Amapá state (Macapá-MAC and Oiapoque-OIA). At each season, in *ca.* 1 km² area around the central point of each locality, sampling was performed by using ovitraps left for one week in the selected areas (Table I) and by collecting all available larvae, days when ovitraps were installed and removed. *Ae. aegypti* eggs and larvae were reared to the adult stage in an insectary with standardised controlled conditions. F0 generation females were used for genotyping.

**TABLE I:** Summary of the date, site and size of the samples of *Aedes aegypti* analysed in the present study

Country	Date of collection	Sampling site (Acronym)	N Microsatellites	N *kdr* at both loci
French Guiana	December 2013	Cayenne (CAY13)	46	50
		Saint Georges (SGO13)	46	50
	May-June 2014	Cayenne (CAY14A)	46	30
		Saint Georges (SGO14A)	46	43
	December 2014	Cayenne (CAY14B)	36	35
		Saint Georges (SGO14B)	36	33
Brazil	May-June 2014	Macapá (MAC14A)	46	-
		Oiapoque (OIA14A)	46	-
	*December 2014-January 2015	Macapá (MAC14B)	-	35
		Oiapoque (OIA14B)	-	35

*: data from Costa.^36^

*Microsatellites genotyping* - DNA was extracted with ZR Tissue and Insect DNA Miniprep kit (Zymo Research, Irvine, CA, United States) for samples OIA14B and MAC14B (Table I), and with a Chelex resin prepared at 5% for the remaining samples. DNA concentration was measured on a NanoDrop® spectrophotometer (ND-1000) and samples were stored at -20ºC.

DNA extracted from 348 mosquitoes of eight sampling points (Table I) was genotyped for 13 microsatellite loci: A1, AC2, AC4, AC7, AG1, AG2, AG4, AG5, B2, CT2,^18^ ^,^ ^19^ 12ACG1, 88AAT1, 201AAT1,^20^ following PCR conditions adapted from the protocols described in the above cited papers. We performed single polymerase chain reaction (PCR) reactions per locus, and mixed the products for fragment analysis. The resulting products were processed for fragment analysis at the DNA Analysis Facility at Yale University, using GS 500 Rox internal size standard (Applied Biosystems, Foster City, CA, United States). The output data from the fragment analysis service was imported and analysed with Genemarker® (Softgenetics, State College, PA, USA).

Data for this study are available upon request.

*Microsatellite Data Analysis* - In order to evaluate the neutrality of loci, we looked for outlier loci using the Dfdist approach with 50.000 simulations in the LOSITAN program.^21^ To increase the reliability of the estimates, we used the options “Neutral mean Fst” and “Force mean Fst”, following the authors’ recommendations.

The number of alleles observed (A) and allele richness (R) based on a minimum sample size of 36 individuals (72 genes) were obtained with HP-RARE.^22^ Estimates of expected heterozygosity (He) were calculated in FSTAT v 2.9.3.2.^23^ Hardy-Weinberg equilibrium (HWE) tests were performed using the software ARLEQUIN 3.5.^24^ Linkage disequilibrium (LD) between pairs of loci was tested using the log likelihood ratio statistic available in GENEPOP v. 4.2.^25^ To test for the presence of null alleles, we used the software MICRO-CHECKER.^26^

Ovitraps may collect several eggs from the same mosquito female oviposition, which may result in sampling of sibling individuals. To ensure that we did not violate assumptions of independent genotypes, we used the maximum-likelihood method implemented in ML-RELATE^27^ to calculate proportions of related individuals within samples. For each pair of individuals, log-likelihood estimates are calculated for four relationship categories: unrelated, parent-offspring, full-siblings, and half-siblings.

The extent of genetic differentiation among temporal and geographical samples was quantified by pairwise Rst based on the stepwise mutation model SMM.^28^ Estimates of Slatkin’s linearised Rst^29^ were tested for correlation with pairwise measures of geographic distance (Google Maps) using Mantel’s tests; significance was calculated by permutation tests (1000 replicates) performed in ARLEQUIN.

In order to compare temporal and spatial continuity across samples, the number of migrants (Nm) was estimated using the private allele method from GENEPOP; which uses rare alleles to estimate gene flow and assumes that they have reached a quasi-equilibrium state.^30^

Bayesian clustering analysis, as implemented in STRUCTURE 2.3.4,^31^ was performed to assess population structure in two datasets. The first dataset comprised only the samples genotyped for 13 loci in this study. Twenty independent runs were performed for each value of K (K = 1 to 8). The second dataset involved a comparison with other American populations,^13^ by combining our data with the microsatellite dataset available in VectorBase.org (Project ID VBP0000176) and using eight common loci (AC2, AC4, CT2, AG1, AG2, AG5, A1, B2). Ten independent runs were performed for each value of K, from 1 to the maximum number of populations analysed. For all STRUCTURE analyses, 100,000 burn-in steps and 500,000 iterations were run, using an admixture model, without prior information on sampling location and assuming independent allele frequencies among populations (λ was set at 1). The most likely value of K was determined with STRUCTURE HARVESTER.^32^ Results were visualised using CLUMPAK.^33^

Factorial correspondence analysis over the initial populations was performed based on pairwise allelic differences using GENETIX v4.03.^34^This method allows for graphically representing multi-locus genetic distances in two- or three dimensions so that the relationships between populations are determined by the way individuals cluster in the dimension plot.

Single-sample estimates of current effective population size (Ne) were calculated by the bias-corrected LD method described by Waples and Do, as implemented in NeEstimator v.2.^35^ Because rare alleles may bias LD *N*e estimates, alleles with frequency below 0.05 were not considered.

Sequential Bonferroni corrections were used to adjust critical probability values for multiple tests to minimise type I errors.

*Genotyping of kdr mutations* - We have genotyped the 1016 and 1534 sites of the voltage gated sodium channel for the specific variations 1016 Val^+^ or Ile^*kdr*^ and 1534 Phe^+^ or Cys^*kdr*^ using a TaqMan (ThermoFischer, Waltham, MA, United States) allelic discrimination assay for samples from French Guiana^5^ and Brazil^9^ with primers, probes and conditions described therein. As both genotyped sites are in the same gene, we considered variations from the two reactions in order to configure the expected alleles: 1016V+ 1534F (‘S’), 1016V + 1534C (‘R1’), 1016I + 1534C (‘R2’) and 1016I + 1534F (‘R3’), as previously reported.^5^ ^,^ ^9^ The combination of these alleles can result in 10 possible genotypes: SS, SR1, SR2, SR3, R1R1, R1R2, R1R3, R2R2, R2R3 and R3R3. For each population, genotype frequencies were calculated by the number of individuals with a respective genotype above the total number of insects successfully genotyped for both sites. The allelic frequencies were obtained by the calculation (n heterozygotes + 2n homozygotes / 2N), where n is the number of individuals with the respective allele and N the total amount of insects successfully genotyped in that population. It is of note that mosquitos genotyped as 1016V/I+1534F/C cannot be discriminated between SR2 and R1R3, and therefore these genotypes were pooled when the allele R3 was present.

## RESULTS

Microsatellite data analysis

*Genetic diversity* - The analysis with Dfdist showed no evidence of outlier loci and all subsequent analyses were thus performed using the 13 loci. The 13 microsatellite loci were polymorphic with the number of alleles ranging from two (AC4) to 19 (AG2). The mean allelic richness over loci varied between four and five. The summary statistics for the 13 loci and the eight populations is presented in Supplementary data (Table I). A total of 12 out of 104 (11%) population by locus comparisons deviated significantly from HWE [Supplementary data (Table I)]. All deviations were caused by heterozygote deficit. After MICROCHECKER analysis, null alleles were suspected in four loci (A1, AG2, AC4, 12ACG1). However, none of the loci consistently deviated from HWE in all the population samples. Indeed, in populations of CAY14B and SGO14B no loci showed evidence for null alleles. As microsatellites used in this study have been extensively validated in previous studies^11^ ^,^ ^18^ ^,^ ^20^ deviations from HWE were associated with null alleles, but at frequencies that did not affect the assessment of population structure. Similarly, 30 out of 624 (4.8%) pairwise comparisons of loci showed significant LD. However, no pair of loci was consistently linked across populations. Therefore, we kept all loci in subsequent analyses. At each population, more than 76% of sampled individuals were unrelated [i.e., no alleles among pairs of individuals were identical by descent; minimum = 76.9% in OIA; maximum = 83.9% in SGO14A, Supplementary data (Table II)].

*Genetic differentiation* - The mean pairwise genetic differentiation over temporal samples measured by Rst (Rst = 0.011) was four times lower than Rst over all populations (Rst = 0.047), and six times lower than the mean pairwise Rst over different spatial samples collected in the same season (May-June 2014: 2014A) (Rst = 0.066). None of the pairwise Rst estimates between temporal samples was significant (Table II). A significant correlation was found between genetic and geographic distances (r = 0.81, p = 0.02), suggesting isolation by distance.

**TABLE II:** Pairwise estimates of Rst (below diagonal) and estimates of number of migrants after correction for size (Nm) among temporal and spatial samples of *Aedes aegypti* (above diagonal)

	CAY 13	CAY 14A	CAY 14B	SGO 13	SGO 14A	SGO 14B	OIA 14A	MAC 14A
CAY13	-	*11.7*	*2.6*	1.1	*-*	*-*	*-*	*-*
CAY14A	0.004	*-*	*3.4*	*-*	1.0	*-*	1.7	0.8
CAY14B	0.029	0.005	-		*-*	1.7	*-*	*-*
SGO13	0.017	0.011	-0.001	-	*10.7*	*4.0*	*-*	*-*
SGO14A	0.036	0.017	-0.011	0.003	-	*3.2*	2.8	0.9
SGO14B	0.061^*^	0.043	0.043^*^	0.006	0.019	*-*	-	*-*
OIA14A	0.090^*^	0.044^*^	0.032	0.050^*^	0.027	0.069^*^	-	1.1
MAC14A	0.104^*^	0.100^*^	0.096^*^	0.100^*^	0.097^*^	0.117^*^	0.108^*^	-

Nm: the comparisons between temporal samples of the same geographic location are presented in *italic*, while comparison between contemporary geographic locations are presented in **bold**. *: significant Rst values after Bonferroni correction.

*Population connectivity and temporal changes* - Temporal connectivity was considered equivalent to connectivity among neighbor populations. Thus, the number of migrants (Nm) was eight (3-12) among the three temporal samples from CAY and 10 (3-11) from SGO (Table II). While across the four spatial samples collected in the same season (2014A), Nm was two, ranging from one (between MAC and CAY) and three (between the neighbor trans-border villages of SGO in French Guiana and OIA in Brazil). These results were consistent with genetic differentiation results. The one-sample estimates of current Ne varied between 11.1 in CAY14B and 55.1 in SGO14B (Table III). Most estimates showed overlapping 95% confidence intervals indicative of no significant differences in Ne among samples. The exceptions were SGO14B and MAC14A. In Cayenne, Ne levels showed high temporal stability.

**TABLE III:** Single-sample estimates of current Ne for *Aedes aegypti*

Population	Ne	CI
CAY	13.7	10.5 - 17.8
CAY14A	12.2	9.2 - 15.9
CAY14B	11.1	8.1 - 15.2
SGO	15.1	11.5 - 19.9
SGO14A	22.2	16.3 - 31.2
SGO14B	55.1	27.7 - 232.2
OIA14A	15.2	11.0 - 21.2
MAC14A	32.0	21.0 - 53.8

Ne: current effective population size based on the bias-corrected LD method; CI: parametric 95% confidence interval.

**Fig. 2:**
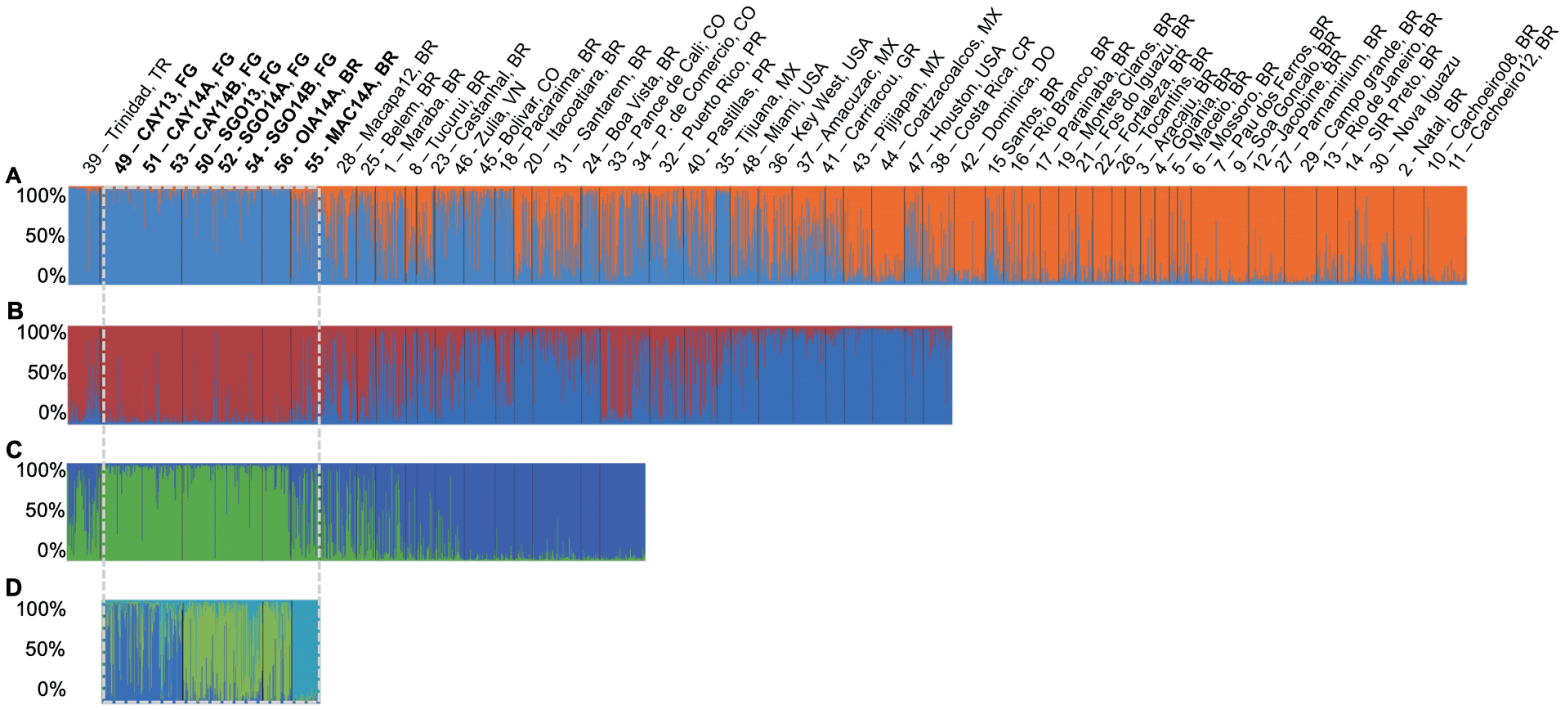
Bayesian clustering analysis by STRUCTURE of *Aedes aegypti*. The multilocus genotype of each individual is represented by a bar. Clusters (K) are represented by different colours and the proportion of each colour in the bar represents the probability of assignment (Q) to each cluster. (A) Analysis of 55 American populations (N = 2,224) with eight loci, sorted by locations and countries. (B) Analysis of the 33 populations (N = 1,409) that were assigned to the blue cluster in (A). (C) Analysis of 15 populations (N = 629) that were selected from (B). (D) Analysis of the eight samples (N = 348) genotyped for 13 loci in this study. These samples are framed by the white dashed line in A, B and C. Sample code numbers are: 1- Marabá, BR; 2- Natal, BR; 3- Aracaju, BR; 4- Goiânia, BR; 5- Maceió, BR; 6- Mossoró, BR; 7- Pau dos Ferros, BR; 8- Tucuruí, BR; 9- São Gonçalo, BR; 10- Cachoeiro_2008, BR; 11- Cachoeiro_2012, BR; 12- Jacobina, BR; 13- Rio de Janeiro, BR; 14- São José do Rio Preto, BR; 15- Santos, BR; 16- Rio Branco, BR; 17- Parnaíba, BR; 18- Pacaraima, BR; 19- Montes Claros, BR; 20- Itacoatiara, BR; 21- Foz do Iguaçu, BR; 22- Fortaleza, BR; 23- Castanhal, BR; 24- Boa Vista, BR; 25- Belém, BR; 26- Tocantins, BR; 27- Parnamirim, BR; 28- Macapá_2012, BR; 29- Campo Grande, BR; 30- Nova Iguaçu, BR; 31- Santarém, BR; 32- Puerto Rico, PR; 33- Pance de Cali, CO; 34- Paso de Comercio Cali, CO; 35- Tijuana, MX; 36- Key West, USA; 37- Amacuzac, MX; 38- Costa Rica, CR; 39- Trinidad, TR; 40- Patillas, PR; 41- Carriacou, GR; 42- Dominica, DO; 43- Pijijapan, MX; 44- Coatzacoalcos, MX; 45- Bolivar, CO; 46- Zulia, VN; 47- Houston, Usa; 48- Miami, USA; 49- CAY13, FG; 50- SGO13, FG; 51- CAY14A, FG; 52- SGO14A, FG; 53- CAY14B, FG; 54- SGO14B, FG; 55- MAC14A, BR; 56- OIA14A, BR. Details about the samples 1-48 are described in Kotsakiozi et al.^13^ and Monteiro et al.^14^

**Fig. 3:**
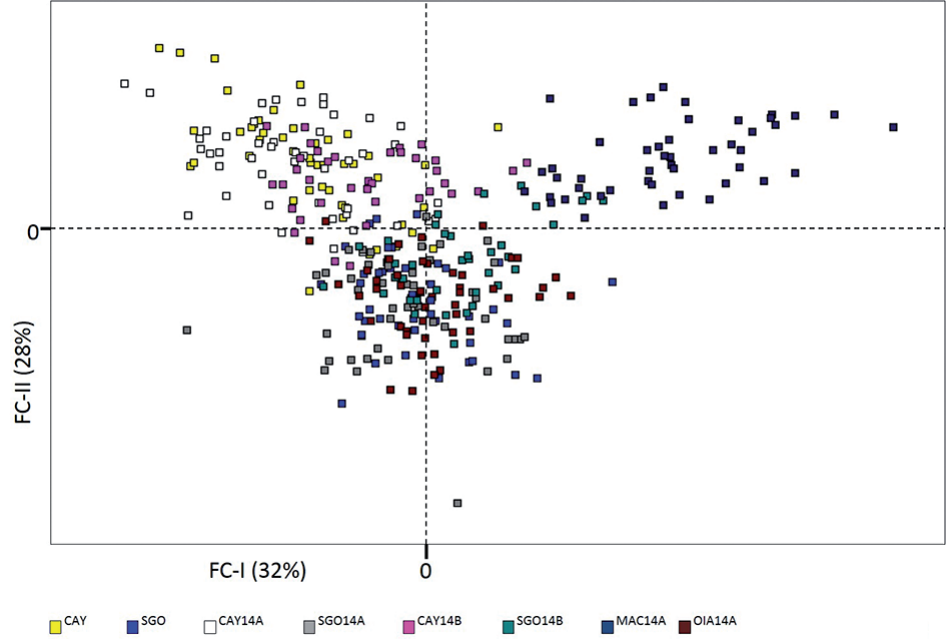
projection of 348 individual microsatellite genotypes of *Aedes aegypti* on the main axes of Factorial Component Analysis. Each colour corresponds to a sampled population as in legend. Inertia percentage values are presented for each factorial component (FC-I and FC-II).

*Population structure* - Finally, the clustering analysis of the 13 loci dataset for eight samples of French Guiana and Amapá state in Brazil identified an optimal number of clusters of K = 3 (Fig. 2D), corresponding to: 1- temporal collections from CAY in green, 2- temporal collections from SGO and OIA in blue, and 3- MAC in yellow. Similarly, Fig. 3 illustrates the position of the 348 genotyped *Ae. aegypti* onto a factorial space based on microsatellite allele frequencies. In the first axis of variation (32%) MAC and CAY appear as the most differentiated populations, while the individuals from the different temporal samples of SGO are mixed with the individuals from OIA.

*Kdr mutations* - Among all specimens, 321 individuals were successfully genotyped for the 1534 codon, 325 for the 1016 codon of the voltage-gated sodium channel and both loci were obtained for 311 of them. The resistant mutation 1016I was absent from MAC but present at high proportions in OIA (67%)^36^ and in French Guiana: CAY (79-94%) and SGO (79-83%) [Supplementary data (Table III)]. On the other hand, the mutant variation 1534C was close to fixation in CAY (96-100%) and present at high frequencies in SGO (77%-80%), OIA (84%) and MAC (90%).

**Fig. 4:**
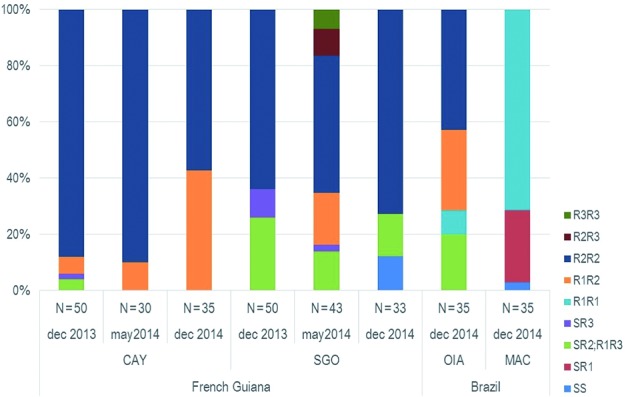
cumulative histogram of genotype frequencies per population. Based on Linss et al.,^9^ ten genotypes from the combined results of the two codons 1016 and 1534: SS (V/V+F/F), SR1 (V/V+F/C), R1R1 (V/V+C/C), SR3 (V/I+F/F), SR2 or R1R3 (V/I+F/C), R1R2 (V/I+C/C), R3R3 (I/I+F/F), R3R2 (I/I+F/C), R2R2 (I/I+C/C). N: Number of individuals screened for each genotype.

The *kdr* genotypic frequencies are presented in Fig. 4. Genotype distributions show a clear distinction between MAC and all the other populations. There was a prevalence of R1 in MAC as either homozygote or heterozygote with S. In the populations from French Guiana and OIA from Brazil genotypic diversity was higher and there was a higher proportion of the double-mutant R2 allele. We have also detected a few R3 allele individuals mainly in SGO [Fig. 4, Supplementary data (Table IV)].

## DISCUSSION

The present study in the Amazonian trans-border region between Brazil and French Guiana revealed high levels of gene flow between the closest cross-border samples of Saint-Georges in French Guiana and Oiapoque in Brazil, showing that the Oyapock River does not act as a physical barrier to mosquito dispersal. The mosquito populations in French Guiana were temporally stable, so natural seasonal factors do not seem to be affecting gene flow and the insecticide resistance patterns in this area. Macapá, the most distant city, was also the most differentiated population for both microsatellites and *kdr* markers. Our results suggest the presence of two genetic groups. In relation with previous knowledge on re-colonisation,^13^ they let us hypothesised that *Ae. aegypti* re-invasion in that area may have occurred through two routes. They also suggest that differences in vector control strategies may have influenced the distinct resistant allele distribution of *Ae. aegypti* from French Guiana and Brazil.

Genetic temporal changes in mosquito populations may occur due to fluctuations in population size, followed by recolonisation or genetic drift events. Such population changes may be human-driven (ex: vector control)^10^ or natural due to seasonal environmental factors like it was reported for *Anopheles* mosquitoes such as in Saint Georges de l’Oyapock.^37^ This was not the case for *Ae. aegypti* from Cayenne or Saint Georges de l’Oyapock, where Nm and Ne estimates were consistent with relative stable temporal samples over one year sampling. Populations at each collection site remained in the same genetic group over time and none of the pairwise comparisons among temporal samples was significantly differentiated. Due to its urban nature associated with artificial breeding sites, *Ae. aegypti* is probably less affected by natural seasonal factors. Similar findings have been reported in Botucatu (São Paulo state, Brazil) where the Ne remained stable over the years, irrespective of the large variation in mosquito abundance.^12^

The pyrethroid-based vector control undertaken in French Guiana during 2014 seems to have had minimal impact in the vector’s effective population size. This agrees with the elevated frequency of *kdr* mutations found in the territory. In addition, local elimination of mosquitoes may be rapidly followed by re-colonisation from neighboring areas that are genetically similar.^10^ However, this situation may have changed during 2015. In the beginning of that year there was a dramatic increase of chikungunya cases, followed by intensification of vector control campaigns and permission to use a different insecticide, the OP malathion.^3^ Additional genetic analyses with post-intervention samples would be required to assess the impact of vector control measures during the epidemic.

The levels of genetic diversity were similar among our samples and comparable with most South American populations.^14^ ^,^ ^15^ ^,^ ^33^ Concerning the samples analysed in the present study, we found a significant correlation between genetic and geographic distances suggesting isolation by distance. Both Bayesian clustering and Factorial Component analysis agreed with this pattern. The most distant localities (Cayenne and Macapá) showed the highest differentiation comparing to the cross-border localities that were much less differentiated, in spite of the Oyapock River separating them. The passive movement of *Ae. aegypti* between both sides can be driven by eggs or larvae remaining in cargo and goods transported by boats,^38^ which cross intensively the river in a daily basis. Our results show that the Oyapock River has not been a strong physical barrier to mosquito dispersal. Furthermore, the recently opened binational bridge will increase the contact between border villages, thus potentially intensifying human-mediated mosquito movement.

It has been recognised that eradication of *Ae. aegypti* in the middle of the 20th century had not been fully achieved in Venezuela, Suriname, Guyana, south of USA and a few Caribbean countries.^14^ These refuge areas may have been the source for the re-colonisation of the American continent during the 1960’s and 70’s, starting with neighboring countries. Northern Brazilian populations are likely to have originated from Venezuela as early as the 1970s whereas southern Brazilian populations seem to have derived more recently from northern Brazilian areas.^13^

When we combined our results with other microsatellite data from South America, we confirmed that our study samples grouped with the above mentioned northern populations,^13^ ^,^ ^14^ showing higher similarity with samples from Trinidad Island and from the state of Pará (neighboring to Amapá state to the East). It is likely that the early re-colonisation of *Ae. aegypti* in 1959 of French Guiana had its origins on the west neighbor country Surinames,^3^ for which we have no samples available to compare. However, our results showed the clustering of French Guiana samples and Oiapoque in Brazil with the island of Trinidad, which may indicate this island as another likely source of re-invasion. On the other hand, the most genetically differentiated sample of Macapá in Brazil grouped with another sample from that city collected in 2012, other cities of Pará and Venezuela.^13^ ^,^ ^14^ ^,^ ^15^ These results suggest a common origin of re-colonisation for the populations of French Guiana and Oiapoque in Brazil, possibly deriving from the Caribbean or an un-sampled population (e.g. Suriname), and a different source for Macapá, in line with a Venezuelan origin of northern Brazilian populations.

Besides the diverse re-invasion histories, differences in vector control should significantly contribute to the shaping of the genetic structure of the studied populations. *Kdr* genotype distributions show a clear distinction between Macapá and all the other populations. In Macapá there is an abundance of R1 alleles (associated with 1534C *kdr*), while in the populations from French Guiana and Oiapoque from Brazil there is higher genotypic diversity and a higher proportion of R2 alleles (two combined resistant mutations, and thus associated with higher levels of resistance.^7^

In Brazil, there is a regional distribution pattern with R1 alleles dispersed throughout the country, while R2 ones are more frequent in central and southeastern areas.^9^ In this country OPs have replaced DDT for some decades (1960’s-2000), when these were also substituted by PYs for adult control.^4^ However, in the southern state of São Paulo (SP) the use of PYs started a decade earlier. In SP, selection pressure after ten years produced high resistance levels to PYs in adults and low levels of resistance to OPs in larvae. While in the Northeast region, where OPs were used over a long period for both larvae and adult control, with PYs only being introduced for adult control in 1999, there were higher resistance levels to OPs in both larvae and adults, and higher susceptibility to PYs in adults by the time of the introduction of this group of insecticides.^4^

Furthermore, the Amazonian states, including Amapá, have been the most affected by endemic malaria, accounting for more than 90% of the cases in Brazil.^39^ Given this large malaria burden, intense *Anopheles* control should be expected. In the Amazonian states, malaria vector control has been implemented over time with DDT in indoor residual spraying until 1997, and PYs since 2000. In 2007, PYs have also been used for insecticide-treated nets distributed free of charge to all age groups.^39^

In addition, the use of domestic insecticides, generally based on PYs, are likely a significant source of the growing selection pressure for *Ae. aegypti* resistant populations in Brazil.^4^

In French Guiana, the pattern of *Ae. aegypti* control is similar to Amapa state with the use of DDT, then OPs until 2011, followed by deltamethrin (PY). However, malaria control had occurred in parallel for which DDT was the main insecticide until 1992, when PYs were introduced for both residual spraying and insecticide-treated nets.^3^ PYs are largely sprayed against mosquito pest all year round. Insecticide pressure of DDT and deltamethrin had been continuous for decades in French Guiana. *Kdr* mutations are associated with cross-resistance to both insecticides. Samples collected in Cayenne 2011 were characterised as highly resistant to deltamethrin.^5^ These studies revealed *kdr* genotype proportions very close to the ones we found in 2013-2014, with the predominance of the R2R2 and R1R2 genotypes. Our samples from Saint Georges were more diversified than Cayenne showing besides R2R2 other genotypes at lower frequency (SS, SR2/R1R3, SR3, R1R2, R2R3 and R3R3). Like for the microsatellite data, Oiapoque was similar to Saint Georges with R2R2, R1R2 and SR2/R1R3 genotypes, but with R1R1 genotype that was absent from the Guyanese samples. On the other hand, this was the dominant genotype in Macapá, where SR1 and SS were also observed in lower frequencies. The *kdr* pattern of Macapá was similar to the one reported for cities in the neighbor state of Pará in 2011.^4^

Our results show distinct *kdr* patterns for Cayenne and Macapá likely associated with their different insecticide use history, and an admixture zone between these two patterns in Saint Georges and Oiapoque. This admixture zone includes a Guyanese city and a Brazilian city where insecticide use patterns were not synchronised. This probably results from the above-mentioned gene flow detected in neutral markers, and also to a possible impact of local *Anopheles* vector and pest mosquito control campaigns.

*In conclusions* - The patterns of cross-border gene flow between *Ae. aegypti* populations and consequent dispersal of insecticide resistance genes observed in this study highlights the need for transnational cooperation in order to monitor and prevent cross-country transportation of vectors (and pathogens). Such cooperation would provide the chance for concerted initiatives that more broadly protect human health through development of preparedness plans. In light of the present results, it is recommended that French and Brazilian authorities adopt common insecticide resistance management plans in this trans-border region.
