# Supplementary material for: Liaisons dangereuses: cross-border gene flow and dispersal of insecticide resistance-associated genes in the mosquito Aedes aegypti from Brazil and French Guiana
Source: Mem Inst Oswaldo Cruz. 2019 Sep 23;114:e190120. doi: 10.1590/0074-02760190120 (PMC6759281; doi:10.1590/0074-02760190120)
Supplement: Supplementary file 1 [file 1678-8060-mioc-114-e190120-s.pdf]

TABLE I

Summary statistics for 13 loci from the eight populations sampled for *Aedes aegypti*

Date of collection	Sampling site		AC2	AC4	CT2	AG1	AG2	AG5	A1	B2	AC7	AG4	12ACG1	88AAT1	201AAT1	All loci
December 2013	Cayenne CAY N = 46	A	3	2	3	4	10	7	4	3	3	6	2	11	6	5
		R	3.0	2.0	3.0	4.0	9.5	6.8	4.0	3.0	3.0	6.0	2.0	10.7	5.7	4.8
		He	0.400	0.269*	0.654	0.604	0.768	0.672	0.619	0.183	0.388	0.576	0.307*	0.868	0.434	0.519
		Fis	-0.031	1.000	-0.064	-0.030	-0.189	-0.197	0.086	-0.071	-0.065	0.018	0.646	-0.002	0.048	0.024
	Saint Georges SGO N = 46	A	3	2	3	5	10	5	4	3	3	6	2	13	6	5
		R	2.8	2.0	3.0	4.6	9.8	4.8	4.0	2.8	3.0	5.6	2.0	12.1	5.9	4.8
		He	0.394	0.294*	0.661	0.654	0.866*	0.697	0.628	0.124	0.382	0.439	0.291	0.831	0.704	0.536
		Fis	-0.212	1.000	-0.076	0.116	0.333	-0.116	-0.108	-0.049	-0.082	0.009	0.254	-0.046	0.166	0.071
May-June 2014	Cayenne CAY14A N = 46	A	3	2	3	4	10	6	4	3	3	6	2	10	6	5
		R	3.0	2.0	3.0	4.0	9.1	6.0	4.0	2.8	3.0	6.0	2.0	9.9	6.0	4.7
		He	0.404	0.264*	0.619	0.624	0.759	0.687	0.595*	0.124	0.362	0.569	0.289*	0.813	0.701	0.524
		Fis	-0.267	1.000	0.067	0.059	-0.174	0.019	0.389	-0.049	-0.082	-0.210	0.764	0.064	0.081	0.076
	Saint Georges SGO14A N = 46	A	3	2	3	4	12	6	4	3	3	5	2	11	5	5
		R	3.0	2.0	3.0	4.0	11.3	5.8	4.0	3.0	3.0	5.0	2.0	10.5	4.8	4.7
		He	0.465	0.322*	0.639	0.680	0.859*	0.749	0.631	0.312	0.367	0.467	0.214	0.793	0.700	0.554
		Fis	-0.075	1.000	-0.054	0.233	0.418	-0.190	0.001	0.024	-0.125	-0.024	0.287	-0.069	0.161	0.097
December 2014	Cayenne CAY14B N = 36	A	2	2	4	3	11	6	4	3	3	6	2	10	6	5
		R	2.0	2.0	4.0	3.0	11.0	6.0	4.0	3.0	3.0	6.0	2.0	10.0	6.0	4.8
		He	0.379	0.282	0.649	0.648	0.856	0.668	0.639	0.225	0.508	0.580	0.283	0.819	0.775*	0.562
		Fis	-0.172	0.014	-0.070	-0.044	-0.129	0.059	-0.280	-0.113	-0.275	0.114	0.213	-0.041	0.176	-0.044
	Saint Georges SGO14B N = 36	A	2	2	4	4	10	5	4	2	2	3	2	11	4	4
		R	2.0	2.0	4.0	4.0	10.0	5.0	4.0	2.0	2.0	3.0	2.0	11.0	4.0	4.2
		He	0.281	0.450	0.658	0.580	0.750	0.517	0.528	0.028	0.178	0.217	0.254	0.835	0.749	0.463
		Fis	-0.186	-0.111	-0.225	-0.005	-0.051	-0.181	-0.157	0.000	-0.094	-0.084	0.364	-0.060	0.258	-0.045
May-June 2014	Macapá MAC14A N =4 6	A	3	2	3	4	10	4	3	2	3	5	2	11	4	4
		R	2.8	2.0	3.0	3.8	9.1	3.8	3.0	2.0	3.0	4.8	1.8	10.5	4.0	4.1
		He	0.505	0.372*	0.582	0.593	0.735	0.638	0.451	0.261	0.317	0.393	0.023	0.829	0.661	0.489
		Fis	-0.162	1.000	0.140	-0.027	0.260	-0.056	0.325	0.168	-0.029	0.115	0.000	-0.023	-0.349	0.077
May-June 2014	Oiapoque OIA14A N =46	A	2	2	3	4	8	4	3	2	3	5	2	7	6	4
		R	2.0	2.0	3.0	4.0	7.8	4.0	2.8	2.0	3.0	4.8	2.0	7.0	5.7	3.8
		He	0.504	0.414*	0.463	0.553	0.820	0.674	0.503	0.124	0.254	0.563	0.043	0.707	0.709	0.487
		Fis	-0.294	1.000	0.087	-0.062	-0.034	0.012	-0.038	0.650	-0.028	0.073	1.000	-0.045	-0.011	0.056
	All populations	A (mean)	2	2	2	3	8	4	3	2	2	4	2	7	4	3
		A (total)	4	2	5	5	19	8	5	3	4	8	3	18	7	91
		He (mean)	0.417	0.333	0.616	0.617	0.802	0.663	0.574	0.173	0.345	0.476	0.213	0.812	0.679	0.517
		Fis	-0.175	0.757	-0.022	0.039	0.073	-0.082	0.033	0.066	-0.102	0.003	0.451	-0.026	0.064	0.045

N: number of specimens genotyped; A: number of observed alleles; R: allelic richness based on a minimum sample size of 36 individuals (72 genes); He: Expected heterozygosity; *: significant deviation from HWE ($p < 0.0005$) after Bonferroni correction; Fis: inbreeding coefficient of an individual (I) relative to the subpopulation (S) that measures heterozygote deficit within populations.

cc BY

TABLE II

Percent of individuals within each *Aedes aegypti* population assigned to the four relationship categories

Population	U	HS	FS	PO
CAY13	82.0	10.1	5.3	2.5
CAY14A	83.7	8.3	6.3	1.7
CAY14B	81.5	10.0	5.8	2.7
SGO13	83.5	9.7	4.7	2.1
SGO14A	83.9	10.4	3.4	2.3
SGO14B	77.8	12.6	4.6	5.0
OIA14A	76.9	13.5	4.3	5.3
MAC14A	79.9	13.8	3.1	3.2

U: unrelated; FS: full-siblings; HS: half-siblings; PO: parent-offspring.

TABLE III

Frequencies of the *kdr* mutations V1016I and F1534C in the populations of *Aedes aegypti* from French Guiana and Brazil analysed independently

Country	Collection time	Population	site 1016				site 1534			
			N	RR - I/I	SR - V/I	SS - V/V	N	RR - C/C	SR - F/C	SS - F/F
French Guiana	dec 2013	CAY13	50	0.88	0.12	0.00	50	0.94	0.04	0.02
		SGO13	50	0.64	0.36	0.00	50	0.64	0.26	0.10
	may2014	CAY14A	43	1	0	0.00	30	1.00	0.00	0.00
		SGO14A	43	1	0	0.00	50	0.63	0.27	0.10
	dec 2014	CAY14B	35	0.57	0.43	0.00	36	1.00	0.00	0.00
		SGO14B	34	0.70	0.18	0.12	35	0.73	0.14	0.11
Brazil*	dec 2014	OIA14B	35	0.43	0.49	0.08	35	0.80	0.20	0.00
		MAC14B	35	0.00	0.00	1.00	35	0.71	0.26	0.03

N: number of individuals analysed; RR: homozygous resistant; SR: heterozygous; SS: homozygous susceptible; I: isoleucine; V: valine; C: cysteine; F: phenylalanine; *: data from Costa.[36]

TABLE IV

Biallele frequencies for *kd* loci per population of *Aedes aegypti* from French Guiana and Brazil

Country	Collection time	Population	N	S	R1	R2	R3
French Guiana	dec 2013	CAY13	50	0.03	0.03	0.93	0.01
		SGO13	50	0.18	0.00	0.77	0.05
	may2014	CAY14A	30	0.00	0.05	0.95	0.00
		SGO14A	43	0.08	0.09	0.70	0.13
	dec 2014	CAY14B	35	0.00	0.21	0.79	0.00
		SGO14B	33	0.20	0.00	0.80	0.00
Brazil*	dec 2014	OIA14B	35	0.10	0.23	0.67	0.00
		MAC14B	35	0.16	0.84	0.00	0.00

Four alleles were identified '1016V + 1534F' called S, '1016V + 1534C' (1534 *kdr*) called R1, '1016I + 1534 C' called R2 (1016 *kdr*+1534 *kdr*) and '1016I + 1534 F' called R3 (1016 *kdr*) according to Linss et al.[9] N: number of individuals analysed; *: data from Costa.[36]
